# Microbial interactions and differential protein expression in *Staphylococcus aureus* –*Candida albicans* dual-species biofilms

**DOI:** 10.1111/j.1574-695X.2010.00710.x

**Published:** 2010-07-01

**Authors:** Brian M Peters, Mary Ann Jabra-Rizk, Mark A Scheper, Jeff G Leid, John William Costerton, Mark E Shirtliff

**Affiliations:** 1 Graduate Program in Life Sciences, Molecular Microbiology and Immunology Program, University of Maryland – BaltimoreBaltimore, MD, USA; 2 Department of Microbial Pathogenesis, University of Maryland – Baltimore, Dental SchoolBaltimore, MD, USA; 3 Department of Oncology and Diagnostic Sciences, Dental School, University of Maryland – BaltimoreBaltimore, MD, USA; 4 Department of Pathology, School of Medicine, University of MarylandBaltimore, MD, USA; 5 Department of Biological Sciences, Northern Arizona UniversityFlagstaff, AZ, USA; 6 Department of Orthopedic Surgery, Center for Genomic Sciences, Allegheny-Singer Research Institute, Allegheny General HospitalPittsburgh, PA, USA; 7 Department of Microbiology and Immunology, School of Medicine, University of Maryland – BaltimoreBaltimore, MD, USA

**Keywords:** *Candida albicans*, *Staphylococcus aureus*, polymicrobial, biofilm, proteome

## Abstract

The fungal species *Candida albicans* and the bacterial species *Staphylococcus aureus* are responsible for a majority of hospital-acquired infections and often coinfect critically ill patients as complicating polymicrobial biofilms. To investigate biofilm structure during polymicrobial growth, dual-species biofilms were imaged with confocal scanning laser microscopy. Analyses revealed a unique biofilm architecture where *S. aureus* commonly associated with the hyphal elements of *C. albicans*. This physical interaction may provide staphylococci with an invasion strategy because candidal hyphae can penetrate through epithelial layers. To further understand the molecular mechanisms possibly responsible for previously demonstrated amplified virulence during coinfection, protein expression studies were undertaken. Differential in-gel electrophoresis identified a total of 27 proteins to be significantly differentially produced by these organisms during coculture biofilm growth. Among the upregulated staphylococcal proteins was l-lactate dehydrogenase 1, which confers resistance to host-derived oxidative stressors. Among the downregulated proteins was the global transcriptional repressor of virulence factors, CodY. These findings demonstrate that the hyphae-mediated enhanced pathogenesis of *S. aureus* may not only be due to physical interactions but can also be attributed to the differential regulation of specific virulence factors induced during polymicrobial growth. Further characterization of the intricate interaction between these pathogens at the molecular level is warranted, as it may aid in the design of novel therapeutic strategies aimed at combating fungal–bacterial polymicrobial infection.

## Introduction

In nature, most microorganisms are associated with surfaces in multispecies biofilm consortia. A biofilm can be defined as a community of microorganisms embedded in a self-derived polymeric matrix, attached to a surface. In a polymicrobial biofilm where multiple microbial species are closely associated, mutually beneficial interactions may develop. Polymicrobial biofilms are found in nearly every niche in the human body; the oral cavity and gastrointestinal and urogenital tracts exhibit tremendous microbial phylogenetic diversity (Aas *et al.*, 2005; Manson *et al.*, 2008;). Although recent decades have witnessed a surge in the area of biofilm research, relatively little is known about the behavior of communities of mixed microorganisms, particularly fungal–bacterial biofilms. Biofilm-embedded organisms demonstrate a uniquely altered gene expression, and studies have suggested that amplified pathogenic phenotypes may emerge during multispecies interactions (Mastropaolo *et al.*, 2005; O'Connell *et al.*, 2006;). One particular biofilm-mediated microbial association, of medical interest, is that which exists between the prokaryotic pathogen *Staphylococcus aureus* and the eukaryotic pathogen *Candida albicans* (for a review, see Shirtliff *et al.*, 2009).

Methicillin-resistant *S. aureus* (MRSA) is a gram-positive coccoid bacterium that is responsible for a significant and increasing number of hospital- and community-acquired infections worldwide (Klevens *et al.*, 2007). This species possesses a number of virulence factors including adhesins, immunoavoidance factors, toxins, coagulase, and a variety of antimicrobial resistance genes (Gordon & Lowy, 2008). The multiple virulence factors of MRSA, coupled with its inherent ability to resist antibiotic therapy via antibiotic resistance gene expression and biofilm formation, have made this pathogen a significant burden to the medical community (Goetghebeur *et al.*, 2007).

*Candida albicans*, a fungal species commonly colonizing human mucosal surfaces, has long been adapted to the human host. However, under conditions of immune dysfunction, *C. albicans* strains cause recurrent mucosal infections and life-threatening disseminated infections (de Repentigny *et al.*, 2004). Multiple antifungal-resistant forms of *C. albicans* are also being increasingly encountered in the hospital setting (Ramage *et al.*, 2002). As a polymorphic species, *C. albicans* is capable of switching morphology between yeast, hyphal, and pseudohyphal forms, a transition central to its pathogenesis. Once in the hyphal form, host epithelial layers can be pierced, a crucial step in the initiation of candidiasis (Sudbery *et al.*, 2004).

Currently, *S. aureus* and *Candida* spp. are ranked among the top three bloodstream pathogens causing severe morbidity and mortality in hospitalized patients. Not only are *C. albicans* and *S. aureus* responsible for a substantial number of infections independently, there is increasing evidence suggesting that they are commonly associated as coinfecting organisms (Abe *et al.*, 2001; Baena-Monroy *et al.*, 2005;). The clinical outcomes of polymicrobial sepsis compared with monomicrobial sepsis are grave, with significantly higher mortality rates (Pulimood *et al.*, 2002). A study by Klotz *et al.* (2007) examining the incidence of candidal bloodstream infections in hospitals reported an *S. aureus–Candida* spp. co-culture rate of up to 20%.

*Candida albicans* and *S. aureus* have also been coisolated from various mucosal surfaces including vaginal and oral mucosa in a biofilm mode of growth. Although *S. aureus* was thought to be a transient member of the oral microbial communities, increasing evidence from several culturing surveys suggests that it is a common isolate from the oral cavity in healthy children and adults, especially in saliva, supragingival plaque, and on the tongue (Miyake *et al.*, 1991; Smith *et al.*, 2003; Ohara-Nemoto *et al.*, 2008;). More seriously, these pathogens have been coassociated with a number of polymicrobial diseases including ventilator-associated pneumonia, cystic fibrosis, superinfection of burn wounds, urinary tract infections, and denture stomatitis (Ekwempu *et al.*, 1981; Dahlen *et al.*, 1982; Siegman-Igra *et al.*, 1988; Smith *et al.*, 2003; Valenza *et al.*, 2008;). Some of the most compelling evidence for this particular bacterial–fungal interaction was demonstrated through a series of studies by Carlson and colleagues (Carlson, 1983; Carlson & Johnson, 1985;). The findings from these studies demonstrated a 6–70 000-fold decrease in the lethal dose 50% of *S. aureus* when coinoculated intraperitoneally with *C. albicans* in mice compared with single-species infections. Despite the significance of these observations, limited studies have examined the interactions of *C. albicans* and *S. aureus* during biofilm development, their most common infectious mode of growth.

In this study, we elucidated the nature and spatial relationship of the interactions between these two diverse pathogenic species using confocal scanning laser microscopy (CSLM) as they coexist and interact during polymicrobial biofilm growth. We have also characterized proteomic changes specific to polymicrobial culture of this cross-kingdom biofilm using two-dimensional differential in-gel electrophoresis (DIGE) and identified differentially regulated metabolic, stress, and virulence proteins via matrix-assisted laser desorption/ionization time-of-flight/time-of-flight tandem MS (MALDI-ToF/ToF MS) analysis.

## Materials and methods

### Strains and growth conditions

The MRSA hospital-acquired clinical isolate used in all the experiments was obtained from a patient with a biofilm-mediated infection at the University of Texas Medical Branch – Galveston and previously designated as strain M2 (Brady *et al.*, 2006). The well-characterized *C. albicans* lab strain SC5314 was used for all the experiments (Gillum *et al.*, 1984). In addition, *S. aureus* strain Seattle 1945 [containing a plasmid encoding for chloramphenicol resistance and green fluorescent protein (GFP) expression under control of the *sarA* promoter] and the constitutively GFP-expressing *C. albicans* strain CAF2-1 were also used (Morschhauser *et al.*, 1998; Leid *et al.*, 2002;). The following bacterial strains were also used: *Staphylococcus epidermidis* (clinical isolate), *Pseudomonas aeruginosa* (PA01), *Streptococcus pyogenes* (clinical isolate), *Bacillus subtilis* (ATCC #6633), and a laboratory strain of *Escherichia coli* (DH5-α).

For all studies, an aliquot of a glycerol stock of *C. albicans* strain SC5314 or GFP-expressing CAF2-1 was grown and maintained on Sabouraud dextrose agar (BBL, Cockeysville, MD). Cultures were grown overnight in yeast peptone dextrose (YPD) (BBL, Sparks, MD) in an orbital shaker (120 r.p.m.) at 37 °C under aerobic conditions. Yeast cells were harvested and washed twice in sterile phosphate-buffered saline (PBS). Starter cultures of clinical isolates of *S. aureus* (M2), GFP-expressing *S. aureus* (Seattle 1945), *S. epidermidis* (clinical isolate), *P. aeruginosa* (PA01), *S. pyogenes* (clinical isolate), *B. subtilis* (ATCC #6633), and a laboratory strain of *E. coli* (DH5-α) were grown in trypticase soy broth (TSB) (Remel, Lenexa, KS) and incubated overnight at 37 °C. Fresh log-phase bacterial starter cultures were grown by diluting the overnight culture 1 : 100 in fresh TSB for 3 h. Bacterial cultures were then washed twice in sterile PBS. Dual-species biofilms were grown in RPMI 1640 buffered with HEPES and supplemented with l-glutamine (Invitrogen, Grand Island, NY) and 5% heat-inactivated fetal bovine serum (RPMI–FBS) (Hyclone, Logan, UT) or YPD containing 5% FBS medium (YPD–FBS).

### Biofilm growth

*Staphylococcus aureus* was grown as noted above and diluted to an OD_600 nm_ of 0.1. *Candida albicans* overnight cultures were grown as described above and diluted to an OD_540 nm_ of 1.0. Biofilms for protein nucleic acid (PNA)-FISH were grown for 24 h on glass coverslips in polystyrene 6-well plates (Corning, Lowell, MA) in 5 mL of RPMI–FBS. Dual-species biofilms were grown by inoculating wells with 50 μL of both species suspensions. PNA-FISH was performed as per the manufacturer's protocol (Advandx, Woburn, MA) with a Cy3-labeled *C. albicans*/fluorescein isothiocyanate (FITC)-labeled *S. aureus* PNA probe cocktail. Nonadherent cells were removed by washing with PBS before imaging. Fluorescence was captured with a Zeiss LSM 510 (Carl Zeiss, Thornwood, NY) confocal microscope using a × 20 objective and a FITC/Texas Red dual-band filter. In order to confirm the strain-independent interaction of *S. aureus* and *C. albicans*, dual-species biofilms of GFP-expressing strains were grown on glass coverslips in RPMI–FBS supplemented with 10 μg mL^−1^ chloramphenicol. Coverslips were processed for microscopy as described above. Finally, microbial protein samples for proteomic studies were prepared by growing mono- or dual-species biofilms in 6-well polystyrene plates as above in either 5 mL of RPMI–FBS (for experiments with hyphae) or YPD–FBS (for experiments with yeast cells) at 37 °C for 24 h.

### Hyphal–bacterial attachment assay

Hyphae formation was induced by first growing *C. albicans* as described previously on glass coverslips in 6-well plates in 3 mL RPMI–FBS for 4 h. Nonadherent hyphae were removed by gently washing the coverslips in PBS, followed by the addition of 3 mL of fresh RPMI–FBS. Log-phase bacterial cell suspensions were washed in PBS, equalized to an OD_600 nm_ of 0.1, and added to the *C. albicans* biofilms. Plates were placed on a rotary shaker to distribute the bacteria evenly and incubated for 1 h at 37 °C. Following incubation, nonadherent cells were removed by gently washing the coverslips in PBS and then examined using phase-contrast microscopy under a × 100 oil-immersion objective. The total number of bacterial cells per field and attached bacteria per hyphae were counted. Percent attachment was calculated by dividing the number of attached bacteria by the total number of bacteria. A total of 10 random fields per coverslip were analyzed.

### Morphological specificity binding assay

Hyphal and blastospore biofilms were grown as described above in RPMI–FBS or YPD–FBS, respectively. Nonadherent cells were gently removed by washing in PBS. Log-phase staphylococcal cell suspensions were added to the *C. albicans* biofilms, shaken, and incubated for 1 h 37 °C. Following incubation, nonadherent cells were removed by gently washing the coverslips in PBS and then examined using phase-contrast microscopy under a × 100 oil-immersion objective. Attachment rates were calculated by counting the total number of yeast cells or hyphae per field as well as the number of attached *S. aureus* cells. These numbers were divided to calculate the average number of *S. aureus* attached per *C. albicans* cell. A total of 10 random fields per coverslip were analyzed.

### Microbial viability assay

Polymicrobial biofilms were grown on glass coverslips as described previously using *C. albicans* SC5314 and *S. aureus* M2. Coverslips were removed from the incubator after 12, 24, and 40 h of growth. Biofilms were washed briefly in PBS, placed into sterile 6-well plates, and stained using the BacLight LIVE/DEAD viability kit (Invitrogen, Carlsbad, CA) according to the manufacturer's protocol. The BacLight LIVE/DEAD system stains live cells green (Syto9), while dead cells appear red (propidium iodide). Coverslips were then mounted onto glass slides with Vectashield (Vector Laboratories, Burlingame, CA) and processed for CSLM. The spatial arrangement of the polymicrobial biofilm was determined by analysis of confocal *z*-axis image slices using the lsmix software package (Carl Zeiss).

### Proteomic analysis

Plates containing 24-h biofilms were gently shaken on a rotary shaker for 1 min and then the culture supernatants were discarded. To remove the biofilms from the wells, 1 mL of cell wash buffer (10 mM Tris, 5 mM Mg acetate, pH 8.0) supplemented with 3 mM phenylmethanesulfonyl fluoride was added and a cell culture tissue scraper was used to remove attached cells. Cells were then washed twice in cell wash buffer, resuspended in 1 mL lysis buffer (30 mM Tris, 4 M urea, 2 M thiourea, 1% CHAPS), and incubated on ice for 10 min. Cells were then mechanically disrupted in a FastPrep FP120 (ThermoSavant, Holbrook, NY) using 0.1 mm zirconia beads (Biospec Products, Bartlesville, OK) for 30 s, followed by a 2-min incubation on ice; the process was repeated for a total of 10 times. Suspensions were centrifuged for 10 min at 14 000 ***g*** and supernatants were removed and protein was quantified spectrophotometrically using the Advanced Protein Assay Reagent #2 (Cytoskeleton Inc., Denver, CO). Crude protein extracts were precipitated and purified with Perfect-Focus reagent as per the manufacturer's directions (G-Biosciences, Maryland Heights, MO) and stored at −70 °C until used.

Two-dimensional DIGE was performed according to the concepts of O'Farrell and Minden and outlined by Sauer and Camper (O'Farrell, 1975; Sauer & Camper, 2001; Minden, 2007;). Protein labeling was performed using the DIGE system (GE Healthcare, Piscataway, NJ) according to the manufacturer's instructions. To achieve sufficient protein rehydration, 100 μg of each protein sample was resuspended in 150 μL of rehydration buffer (30 mM Tris, 7 M urea, 2 M thiourea, 2.5% CHAPS). Following rehydration, the pH was adjusted to 8.5 with dilute NaOH or HCl as needed. *Candida albicans* proteins were labeled with Cy2, *S. aureus* proteins were labeled with Cy3, and co-cultures were labeled with Cy5 at a ratio of 2 pmol CyDye μg^−1^ protein. Samples were incubated for 30 min on ice and kept protected from light. Following CyDye labeling, 15 μL of 10 mM lysine was added for 10 min to quench excess CyDye. Samples were combined and a final concentration of 35 mM DTT and 1.6% Pharmalyte 3-10 was added. Samples were applied to 24 cm, pH 3–10 (linear) Immobiline Dry-Strips (IPG) (GE Healthcare). Proteins were separated in the first dimension by their isoelectric point using a Multiphor II (Amersham) as per the manufacturer's directions. Before the second dimension, IPG strips were equilibrated and applied to 12% 26 cm × 20 cm sodium dodecyl sulfate-polyacrylamide gel electrophoresis gels. Protein spots were resolved in the second dimension using a Höefer DALT Vertical System and fluorescence was captured using the Typhoon Imager 9400 (GE Healthcare). Following fluorescence scanning, gels were nondestructively silver stained for spot excision (Gharahdaghi *et al.*, 1999). Protein spots that were upregulated in six out of six gels were selected for MALDI-ToF/ToF MS analysis as described previously (Brady *et al.*, 2006).

### Statistics

All studies were performed in triplicate at a minimum. In addition, all cell enumerations were performed on a minimum of 10 fields of view and at least 400 cells. A Student's *t*-test was used to compare microbial numbers, with a *P*<0.05 representing a statistical significance.

## Results

### Hyphal–bacterial attachment assay

**Fig. 1:**
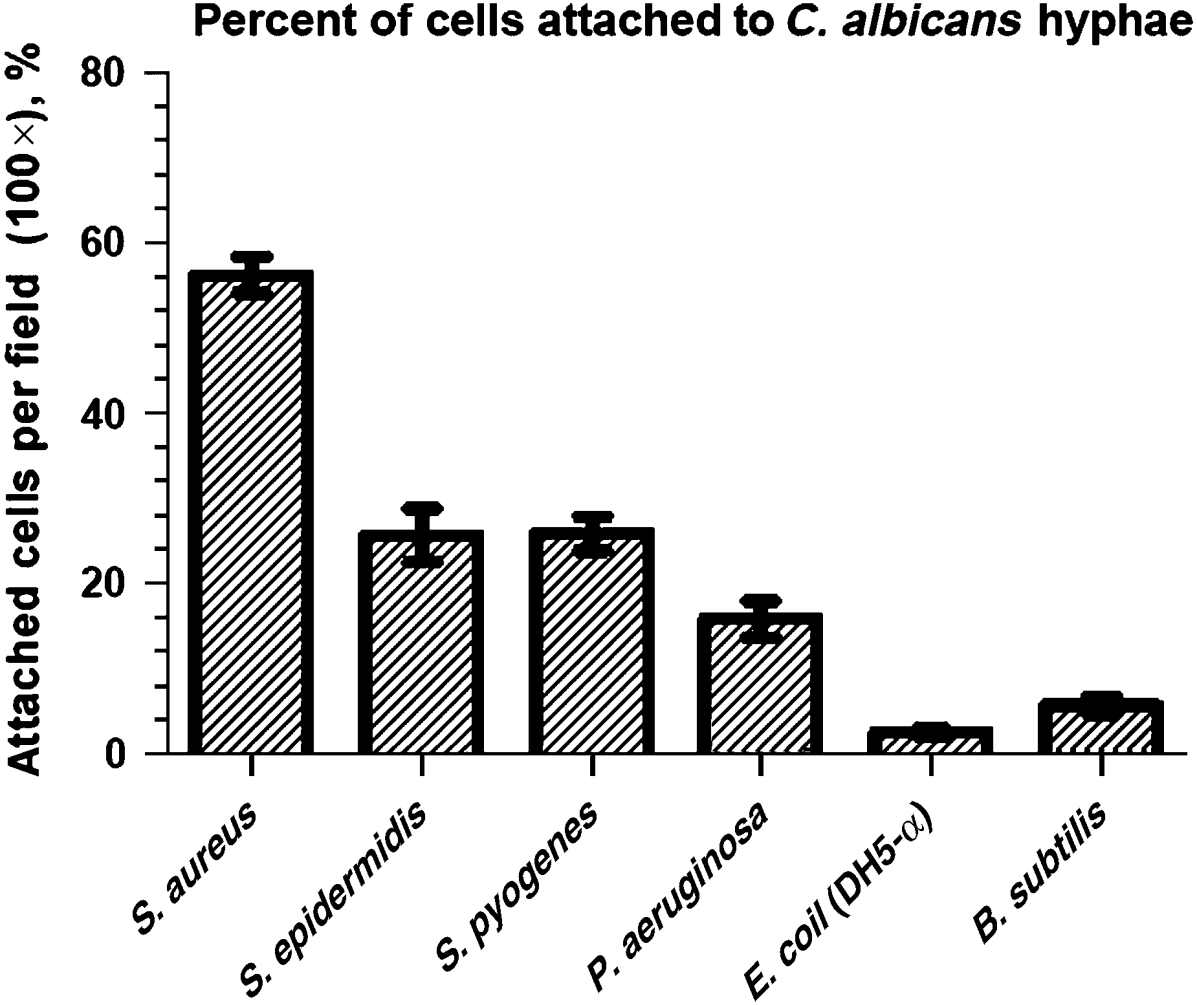
Bacterial attachment assay. *Candida albicans* biofilms were grown for 3 h in RPMI to induce hyphae formation and incubated for 1 h with the following bacteria: *Staphylococcus aureus, Staphylococcus epidermidis, Streptococcus pyogenes, Pseudomonas aeruginosa, Bacillus subtilis, Escherichia coli* (DH5-α). Nonadherent cells were removed by washing and the remaining cells were counted by phase-contrast microscopy. Percent hyphal attachment was assessed by counting the number of bacteria associated with the hyphae divided by the number of total bacteria per field. Ten fields were chosen at random and averaged; the experiment was repeated in triplicate. Error bars represent SD.

In order to assess the potential for hyphal–bacterial interactions, we tested a panel of various bacterial species displaying a wide variety of phenotypes including cell morphology, motility, ecological niche, and Gram stain identity for hyphal interaction. *Candida albicans* biofilms were grown overnight on glass coverslips, washed, and various bacterial strains added for 1 h. Hyphal binding was measured via phase-contrast microscopy as the number of attached bacterial cells to *C. albicans* hyphae divided by the total number of bacterial cells per microscopic field and reported as a percentage (Fig. 1). Percent counts demonstrated that *S. aureus* had the highest hyphal association (56%), followed by *S. pyogenes* and *S. epidermidis* (25%). *Pseudomonas aeruginosa*, a gram-negative motile rod and known hyphae binder, had a hyphal association of (17%), while *E. coli*, also a gram-negative rod, and *B. subtilis*, a gram-positive bacillus, demonstrated the lowest hyphal binding (5.7% and 2.5%, respectively).

### PNA-FISH

**Fig. 2:**
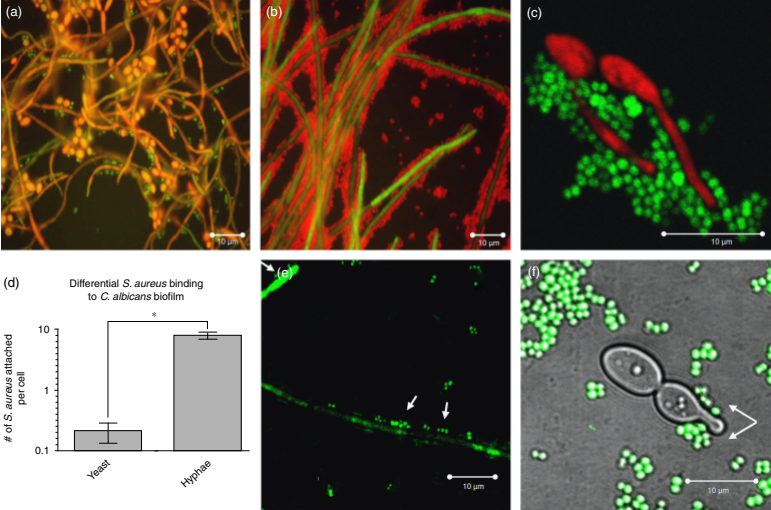
Biofilm architecture of *Candida albicans* and *Staphylococcus aureus* 24-h dual-species biofilm using PNA-FISH and GFP-expressing microorganisms. (a) *Staphylococcus aureus* (FITC-labeled probe, green) has a greater tropism for the hyphal form of *C. albicans* (TAMRA-labeled probe, red) compared with the yeast form. Field of view diameter is 150 μm. (b) An area of *C. albicans* (FITC-labeled probe, green) hyphal biofilm growth is completely covered by *S. aureus* (Cy3-labeled probe, red). (c) A × 63 zoom image showing staphylococci (FITC-labeled probe, green) binding to only the hyphal filaments of *C. albicans* (Cy3-conjugated probe, red). (d) Graph representing the average number of *S. aureus* cells attached per *C. albicans* cell during polymicrobial biofilm growth. Ten fields were chosen at random for counting and the experiment was repeated in triplicate. Error bars represent the SD. (e) *Staphylococcus aureus* (white arrows), expressing GFP under control of the *sarA* promoter, was found to be associated to GFP-expressing *C. albicans* hyphae. (f) *Staphylococcus aureus* (white arrows) demonstrating preferential binding to a *C. albicans* germ tube without binding to the yeast cell. Fluorescence was captured with a × 63 oil-immersion objective and FITC/DICIII, FITC/Texas Red filter sets. Asterisk (^*^) denotes a statistically significant difference at P<0.05.

Because of the strong hyphal binding exhibited, fluorescence microscopy using species-specific PNA-FISH probes was used to visualize the physical interaction between *C. albicans* and *S. aureus* in an *in vitro* dual-species biofilm. Images revealed extensive adherence of *S. aureus* to *C. albicans*, with a preferential association to the invasive hyphal elements of *C. albicans* (Fig. 2a and c). In areas of dense hyphal biofilm growth, *S. aureus* could be seen completely covering *C. albicans* (Fig. 2b). To show the specificity of *S. aureus* for binding the hyphal form of *C. albicans*, polymicrobial interactions were assessed using both hyphae and yeast biofilms. Quantitative counts demonstrated a 30-fold increase in *S. aureus* binding to hyphae as compared with *C. albicans* yeast cells. These observations were confirmed by similar experiments performed using different GFP-expressing strains of *C. albicans* and *S. aureus* where a similar adherence pattern was demonstrated, confirming that this interaction is strain independent (Fig. 2e and f).

### Microbial viability assay

**Fig. 3:**
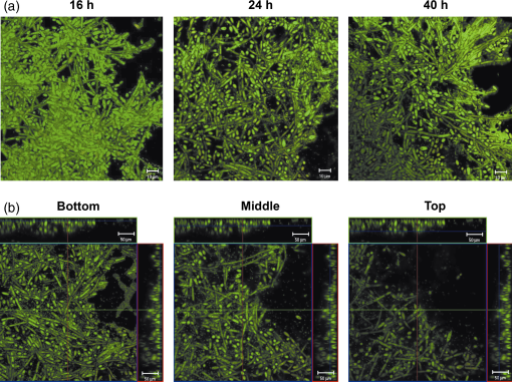
Viability and spatial arrangement in the dual-species biofilm. *Candida albicans*–*Staphylococcus aureus* biofilms were grown for various time points on glass coverslips, stained with BacLight LIVE/DEAD, and processed for CSLM. (a) At all the time points tested, both bacteria and fungi appear healthy as measured by the presence of green fluorescence (Syto9) and the absence of red (propidium iodide). (b) Representative confocal *z*-stack images of a typical 24-h dual-species biofilm demonstrating the presence of *S. aureus* attached to *C. albicans* hyphae throughout the bottom, middle, and top layers.

The BacLight LIVE/DEAD cell viability assay was used to determine whether fungal or bacterial cells were killed during polymicrobial biofilm growth and to assess the spatial arrangement of the biofilm. After 16, 24, and 40 h of growth, both cell types were viable as visualized by green fluorescent staining (Syto9) with an apparent lack of red fluorescence (propidium iodide) (Fig. 3a). In addition to staining for cell viability, the spatial arrangement of the dual-species biofilm was characterized by confocal *z*-stack imaging analysis. Bottom, middle, and top representative *z*-axis image slices from a 24-h polymicrobial biofilm show the presence of *S. aureus* attached to the hyphae of *C. albicans* throughout the entire biofilm architecture (Fig. 3b).

### Proteomic analysis

**Fig. 4:**
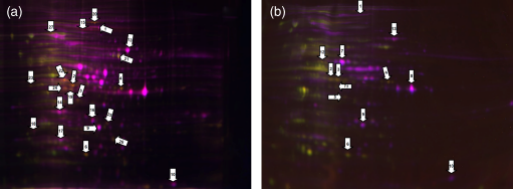
Representative DIGE gel from mono- and dual-species biofilms. Whole-cell lysates, enriched in the cytoplasmic fraction, were obtained from 24-h biofilms. Proteins (100 μg) were differentially labeled with CyDye: *Candida albicans* labeled with Cy2 (blue), *Staphylococcus aureus* labeled with Cy3 (green), dual-species biofilm proteins labeled with Cy5 (red). Proteins were focused in the first dimension on pH 3–10 IEF strips and resolved in the second dimension on 12.5% polyacrylamide gels. (a) Representative gel from staphylococcal–yeast biofilms. (b) Representative gel from staphylococcal–hyphal biofilms.

In order to identify other factors that may lead to increased virulence during coinfection, unfractionated, whole-cell proteins from 24 h *in vitro* biofilms were harvested, purified, and differentially lysine-labeled with NHS-ester CyDyes. Labeled proteins were then combined and subjected to isoelectric focusing and second dimension analysis. Representative gels from either mono- or dual-species biofilms composed of *S. aureus* and *C. albicans* yeast cells (Fig. 4a) or *S. aureus* and *C. albicans* hyphal cells (Fig. 4b) are shown. Spots were considered for MALDI-ToF/ToF MS identification if they were reproducible on six out of six gels. In this global proteomics screen, we identified 27 proteins that were upregulated in the co-culture biofilm. Among these were proteins important for growth and metabolism and others of hypothetical function. Most notable of interest were those proteins implicated in microbial stress and enhanced virulence of both species (Table 1).

**Table 1:** Proteins upregulated in the dual-species biofilm

Spot	MW (Da)	pI	Organism	Identity	Protein name	Peptide matches	Protein score confidence interval (%)	Accession number	Function
(A) Proteins upregulated in staphylococcal–yeast biofilms									
1A	84624.8	5.96	*C. albicans*	Putative mitochondrial aconitate hydratase	Aco1p	19	100	68479387	Carbohydrate metabolism; tricarboxylic acid cycle
14A	21481.5	5.15	*C. albicans*	Similar to heat shock protein 5	Similar to Hsp5	9	100	68469633	Cellular stress response; protein folding
15A	91795.2	6.35	*C. albicans*	Heat shock protein 78	Hsp78p	16	100	31076745	Cellular stress response; protein folding
16A	26893.9	5.74	*C. albicans*	Triosephosphate isomerase	Tpi1p	10	100	7270988	Glycolysis; gluconeogenesis; fatty acid biosynthesis
17A	21960.3	4.98	*C. albicans*	Thioredoxin peroxidase	Tsa1p	7	99.99	68479826	Cellular stress response; antioxidant
18A	49188.7	7.36	*C. albicans*	Metal-binding activator 1	Mac1p	6	85.35	68471167	Copper-binding transcriptional regulator; cellular stress response
19A	95340.7	5.68	*S. aureus*	Alcohol dehydrogenase, iron containing	Adh	15	100	57651152	Carbon utilization; alcohol metabolism
20A	95397.8	5.73	*S. aureus*	Putative aldehyde-alcohol dehydrogenase	AdhE	16	100	49482391	Carbon utilization; putative peroxide scavenger
21A	56138.6	6.02	*S. aureus*	Probable malate:quinone oxidoreductase	Mqo1	21	100	82752186	Carbohydrate metabolism; tricarboxylic acid cycle
22A	37820.9	5.14	*S. aureus*	Ornithine carbamoyltransferase	ArgF	12	100	49484831	Amino acid biosynthesis
23A	35194.1	4.65	*S. aureus*	Pyruvate dehydrogenase complex E1 component β	PdhB	11	100	57651703	Glycolysis; oxidoreductase
24A	35539.3	5.36	*S. aureus*	Carbamate kinase	ArcC1	17	100	49484829	l-Arginine degradation
25A	28737.4	5.87	*S. aureus*	Transcriptional repressor CodY	CodY	8	99.97	15924245	Decreased hemolysin, biofilm, and quorum-sensing function
26A	23092.2	6.08	*S. aureus*	Uracil phosphoribosyl transferase	Upp	10	100	15925102	Pyrimidine metabolism
27A	63331.2	5.2	*S. aureus*	Pyruvate kinase	Pyk	27	100	49483939	Carbohydrate metabolism; glycolysis
(B) Proteins upregulated in staphylococcal–hyphal biofilms									
1B	93865.5	6.07	*C. albicans*	Translation elongation factor 2	Eft2p	4	100	68481380	Protein synthesis
8B	35924.7	6.61	*C. albicans*	Glyceraldehyde 3 phosphate dehydrogenase	Thd1p	15	100	68472227	Carbohydrate metabolism; glycolysis
11B	33026.2	5.4	*S. aureus*	Cysteine synthase	CysK	4	100	82750220	Cysteine biosynthesis
3B	29543.3	5	*S. aureus*	l-Lactate dehydrogenase	Ldh1	9	100	87161566	Growth during nitrosative stress
12B	40322.9	5.2	*S. aureus*	Alanine dehydrogenase 1	Ald1	15	100	21283057	Cell wall synthesis; oxidation reduction
(C) Proteins upregulated in both biofilm conditions									
9A,B	21481.5	5.79	*C. albicans*	Similar to phosphoglycerate mutase	Gpm1p	16	100	68469783	Carbohydrate metabolism; glycolysis
10A,B	17677.9	7.74	*C. albicans*	Cyclophilin type peptidyl-prolyl *cis*–*trans* isomerase	Cyp1p	4	100	68469052	Protein folding; cellular stress response
13A,B	55751.8	6.54	*C. albicans*	Pyruvate kinase	Pyk1p	16	100	68482226	Carbohydrate metabolism; glycolysis
2A,B	36423.8	5.34	*S. aureus*	Alcohol dehydrogenase	Adh	12	100	21282297	Carbon utilization; alcohol metabolism
5A,B	29434.3	5.34	*S. aureus*	30s ribosomal protein S2	RpsB	6	100	57651825	Protein synthesis; stress response
6A,B	18520.5	5.6	*S. aureus*	Similar to universal stress protein family	Similar to UspA1	7	95.7	15924700	Cellular stress response
4A,B	37381.6	6.08	*S. aureus*	Threonine dehydratase	IlvA	16	100	147733998	Amino acid metabolism

## Discussion

Previous studies have identified that *C. albicans*–*S. aureus* intraperitoneal coinfections resulted in enhanced virulence and lethality in a mouse model, but a detailed description of the polymicrobial interactions between these pathogens has remained undefined (Carlson, 1983; Carlson & Johnson, 1985;). To this end, this study was designed to examine the physical interactions and the differential protein expression occurring during *C. albicans*–*S. aureus* polymicrobial biofilm growth.

Because *C. albicans* bacterial binding has been reported previously, the relative *C. albicans* hyphal-binding affinity of other bacteria was evaluated and compared with that of *S. aureus* (Fig. 1). Comparative adherence assays demonstrated that all bacterial species tested, including the more closely related species of *S. pyogenes* and *S. epidermidis*, associated with the hyphae of *C. albicans* significantly less than *S. aureus*. Differences in hyphal binding between various bacteria may be due to differences in surface protein expression or as yet unidentified microbial adhesins. Our assay also demonstrated significantly lower hyphal binding of the well-described *C. albicans*-interacting bacterial species *P. aeruginosa* compared with *S. aureus*. Even with this comparatively lower binding affinity, Hogan *et al.* (2004) have demonstrated that *P. aeruginosa* is capable of killing the hyphae of *C. albicans*, through a process involving the homoserine lactone quorum-sensing molecule, 3-oxo-C12. These observations indicate that, unlike the seemingly mutualistic *C. albicans*–*S. aureus* relationship demonstrated by our studies, the interaction between *C. albicans* and *P. aeruginosa* seems to be antagonistic.

Because of the significantly increased rates of staphylococcal–fungal association identified in the hyphal–bacterial attachment screen, polymicrobial growth was visualized by fluorescence microscopy in order to determine the architecture of the co-culture biofilm. Imaging analysis revealed *S. aureus* adhering to the invasive hyphal filaments of *C. albicans*, but not the round yeast cells (Fig. 2). Confocal *z*-stack imaging showed *S. aureus* to be distributed along the hyphal filaments throughout the entire biofilm architecture (Fig. 3b). These findings differ from the recent findings by Harriott & Noverr (2009) investigating increased drug resistance in polymicrobial biofilms in which *S. aureus* was noted to be attached to hyphal elements mostly in the uppermost layers of the biofilm. Differences in biofilm growth substratum and medium may partially account for these discrepancies. Preference for binding the hyphae of *C. albicans* has been reported in a number of other species, including *S. pyogenes, Acinetobacter baumannii*, and *P. aeruginosa* (Cunningham, 2000; Hogan & Kolter, 2002; Peleg *et al.*, 2008; Bamford *et al.*, 2009;). Many of these previously identified *C. albicans*–bacteria interactions result in fungal and/or bacterial killing during co-culture; however, the *C. albicans*–*S. aureus* interaction described in this study appears to be nonlethal for either organism as measured by the LIVE/DEAD cell viability assay (Fig. 3a). The lack of an antagonistic relationship during polymicrobial growth may have important implications for the enhancement of virulence during coinfection and may partially explain the relatively high rate of co-culture for these organisms. Combined, these important findings highlight the diversity of the interactions that take place between these human pathogens.

In light of the observed extensive association between *S. aureus* and *C. albicans* hyphae, we hypothesized that protein expression may be modulated in the dual-species environment, which could have important implications during coinfection. Hyphal binding may result in altered virulence factor production, augmenting immunoavoidance and/or damage to the host as has been seen in other species (Richard *et al.*, 2002; Sibley *et al.*, 2008;). In order to further characterize the molecular interactions between *C. albicans* and *S. aureus* and to identify the factors that may be responsible for their infectious synergism, a global proteomics approach was utilized; the upregulated proteins identified are listed in Table 1. Among the 27 differentially regulated proteins, some were upregulated either uniquely in the staphylococcal–yeast or staphylococcal–hyphae biofilms or in both co-culture conditions compared with mono-species cultures. These proteins were mainly involved in growth, metabolism, or response to stress including proteins that are inducible upon heat, oxidative, nutrient, and antibacterial stress.

Several stress-related proteins, known to be induced upon heat, oxidative, and antibacterial stress, were found to be consistently upregulated by *S. aureus*, indicating the presence of a stress response by *S. aureus* to both *C. albicans* yeast and hyphal forms (Table 1) (Kvint *et al.*, 2003). Similarly, Cyp1p, a *cis*–*trans* isomerase involved in protein folding and upregulated during oxidative and nutritional stress, was upregulated in *C. albicans* (Dartigalongue & Raina, 1998; Andreeva *et al.*, 1999; Wen *et al.*, 2005;). The upregulation of the uspA-like protein, Cyp1p, and RpsB, a ribosomal protein, is consistent with the findings of upregulated proteins *in vivo* during *Mycobacterium avium* infection and emphasizes that these proteins may be important in resisting heat shock and stress inside the host (Hughes *et al.*, 2007).

Many growth and metabolic proteins in both *C. albicans* and *S. aureus* were upregulated in the mixed biofilm. Contrary to our expectations, however, the majority of the upregulated proteins were present in the staphylococcal–yeast biofilm. Interestingly, *C. albicans* yeast cells demonstrated the upregulation of a significant number of proteins involved in cell stress, including the heat shock proteins, which are highly inducible upon cell stresses including heat, hypoxia, UV exposure, starvation, toxin exposure, and dehydration (Table 1) (Matthews & Burnie, 1992). It is possible that staphylococcal binding to *C. albicans* blastospores within the polymicrobial biofilm may have been evolutionarily selected against under seemingly ‘stressful’ conditions.

In *C. albicans*, Mac1p is a transcription factor that facilitates the uptake of copper. Copper is an important cofactor for a wide variety of cellular enzymes that carry out essential biological processes such as respiration (Marvin *et al.*, 2003). Furthermore, copper is believed to play a detrimental role in protection against oxidative stress, which provides an additional explanation for the observed upregulation of Mac1p. This is corroborated by the observed aforementioned concomitant upregulation of various stress response proteins by *C. albicans* yeast cells. Combined, these findings clearly indicate that the presence of *S. aureus* induces a stress response by *C. albicans*.

Among the proteins of note found to be upregulated in *C. albicans* yeast cells was Tsa1p, a thioredoxin peroxidase important for detoxification after peroxide stress (Urban *et al.*, 2005), and aconitate hydratase, which is highly susceptible to oxidation under stressed conditions (Tang *et al.*, 2002; Matasova & Popova, 2008;). Few proteins were found to be upregulated in the staphylococcal–hyphae biofilm in either organism. In *C. albicans*, the expression of Tef2p, a GTP-binding translational elongation factor important for protein synthesis, was increased (Capa *et al.*, 1998). In *S. aureus*, there was increased expression of alanine dehydrogenase, shown to be involved in the metabolism of alanine and suggested to have a role in bacterial cell wall synthesis (Andersen *et al.*, 1992). In addition, cysteine synthase involved in the biosynthesis of cysteine was also found to be upregulated in *S. aureus. Staphylococcus aureus* mutants deficient in cysteine synthase are more susceptible to oxidative stress, acid, and phosphate-limiting conditions due to the role of cysteine in stress response and survival mechanisms (Lithgow *et al.*, 2004).

Staphylococcal gene products that have been previously shown to play an important role in virulence and pathogenesis were also shown to be differentially regulated under coculture conditions compared with mono-species cultures. In the staphylococcal–yeast biofilm, CodY, a transcriptional repressor of a variety of *S. aureus* virulence factors exhibited increased expression (Levdikov *et al.*, 2006). This protein has was shown to repress PIA-dependent biofilm formation, the production of hemolysins alpha and delta, and proteins involved in the global regulator of virulence, the *agr*-dependent quorum-sensing system (Frees *et al.*, 2005; Majerczyk *et al.*, 2008;). However, CodY was downregulated under the staphylococcal–hyphal biofilm growth conditions. Therefore, decreased CodY expression may enable enhanced toxin-mediated virulence and increased biofilm formation in *S. aureus*.

The virulence-associated l-lactate dehydrogenase 1 (Ldh1), an enzyme involved in the generation of l-lactate during fermentation, was upregulated in the staphylococcal–hyphal biofilm, but not in the staphylococcal–yeast biofilm. Recently, biochemical studies by Richardson and colleagues demonstrated that *S. aureus* Ldh1 is uniquely inducible under nitrosative stress conditions, enabling *S. aureus* to persist in the host in the presence of host-derived nitric oxide. Furthermore, an *S. aureus ldh1* mutant exhibited attenuated virulence compared with wild-type *S. aureus* in a mouse model of systemic infection (Richardson *et al.*, 2008). Closely related staphylococcal species, *S. epidermidis* and *Staphylococcus saprophyticus*, lack Ldh1 and therefore cannot survive under conditions of nitric oxide stress as encountered in host macrophages and neutrophils.

The increased expression of CodY and downregulation of Ldh1 lead us to hypothesize that *S. aureus* may downregulate its virulence while coexisting with *C. albicans* yeast cells at a mucosal surface such as at vaginal, gastrointestinal, or oral tracts as a strategy to remain in a commensal state at these sites, thereby evading detection and clearance by the host immune system. Conversely, candidal germination appears to induce *S. aureus* virulence and biofilm formation capability through the downregulation of CodY expression. The simultaneous increase in Ldh1 expression could potentially combat nitric oxide produced by the host in response to *C. albicans* hyphal invasion (Oliveira *et al.*, 2007). While these proteomics studies are not a comprehensive analysis of the entire proteome, they do demonstrate the plasticity of global protein expression unique to polymicrobial growth. Further experiments to address these polymicrobial-enhanced immunoavoidance and virulence mechanisms, as well as the possible differential expression of cell wall proteins and secreted factors, are warranted and currently underway in our laboratories.

In conclusion, this study characterizes a unique microbial association within the context of a polymicrobial biofilm, in which *S. aureus* binds the hyphal elements of *C. albicans*. In addition, it establishes the presence of a robust and dynamic interaction between two diverse and significant human pathogens by demonstrating the upregulation of several putative virulence factors specific to polymicrobial growth. The findings generated from this investigation will contribute to our understanding of the complex and clinically significant interactions that take place between microbial species as they coexist in the host and during infectious processes. Therefore, continued epidemiologic and laboratory research is needed to better characterize and understand these pathogens in the context of complicated polymicrobial infections, allowing for improved diagnostic and therapeutic strategies in the future.

## Statement

Re-use of this article is permitted in accordance with the Terms and Conditions set out at http://www3.interscience.wiley.com/authorresources/onlineopen.html
